# Cardiac Arrest in an Adolescent Boy Due to the Anomalous Origin of the Left Main Coronary Artery: Emergency Department Perspective

**DOI:** 10.7759/cureus.25771

**Published:** 2022-06-08

**Authors:** Waseem Malik, Hasan Younis, Benny Ponappan, Khalid Bashir, Amr Elmoheen

**Affiliations:** 1 Emergency Department, Hamad Medical Corporation, Doha, QAT; 2 Qatar University Health, College of Medicine, Qatar University, Doha, QAT

**Keywords:** emergency medicine resuscitation, coronary artery angiography, pediatric cardiac arrest, congenital anomalies of coronary arteries, cardiac arrest

## Abstract

Congenital anomalies of the origin of the coronary arteries are rare. Multiple anomalies have been reported where the common one is left circumflex arising from the right sinus of Valsalva (RSV). Other anomalies are a single coronary artery from the left sinus of Valsalva, both coronary arteries originating from RSV, and the left anterior descending from RSV. The left main coronary artery originating from RSV is significant as it carries a high risk of sudden cardiac death. We report here the case of one child who was brought in a cardiac arrest to the emergency department of our hospital. After successful resuscitation and further workup during his stay in the hospital, it was found that he had the left main coronary artery originating from the right Valsalva sinus.

## Introduction

Congenital anomalies involving coronary arteries are rare, ranging from 0.24% to 1.3% [1,2]. The left main coronary artery (LMCA) arising from the right sinus of Valsalva (RSV) is the most critical, with a high risk of sudden cardiac death [2-6]. The other anomalies are left circumflex originating from the RSV or right coronary artery (RCA, most common), left anterior descending (LAD) from RSV, both coronary arteries from RSV, and a single coronary artery from the left sinus of Valsalva [3]. We present a case of a child who had a cardiac arrest and, on computed tomography (CT) angiogram, was found to have an anomalous origin of the LMA from the RSV.

## Case presentation

A 14-year-old adolescent boy was brought by ambulance to the emergency department (ED) in a cardiac arrest. The boy, while playing soccer, felt chest pain and shortness of breath, then he started becoming drowsy, and ambulance services were called. On arrival, the ambulance crew saw that the patient was conscious but drowsy and having labored breathing. While on the way to the ED, the patient had a cardiac arrest. Immediately cardiopulmonary resuscitation (CPR) was started, and the airway was secured. The initial rhythm was ventricular fibrillation (VF), and the patient received defibrillation of 200 joules. Venous blood gases (VBG) analysis during CPR showed potassium of 6.2 mmol/l (the normal range is 3.5-5.3 mmol/l), and hyperkalemic measures were given in the form of glucose-insulin infusion and calcium. After approximately 20 minutes of CPR and receiving six shocks, spontaneous circulation was returned. The patient was admitted to the medical intensive care unit (MICU) for further evaluation. The electrocardiogram (ECG) showed sinus rhythm and peaked T waves in V1 to V3 and widening Q wave, R wave, and S wave (QRS) complex (ECG features of hyperkalemia). Subsequently, ECGs afterward were normal. A bedside echocardiogram showed severe reduced systolic left ventricle (LV) function, severe global hypokinesia of LV, ejection fraction (EF) around 20%, and normal right ventricle (RV) function.

After resuscitation, CT of the brain was done and was normal. His laboratory findings showed increased white blood cell count, creatinine, potassium, and troponin T (Table 1).

**Table 1:** Patient's biochemical parameters in the emergency department (ED) and the medical intensive care unit (MICU) NT-proBNP - N-terminal-pro hormone BNP

Investigations	In ED	In MICU	Normal laboratory range
Blood glucose (mmol/L)	4.7	4.3	3.3-5.5
Troponin T high sensitive (ng/L)	900.10	47.64	0.00-14.0
Hemoglobin (gm/dl)	18	12.6	12-15
Arterial pH (mmol/l)	6.802	7.436	7.35-7.45
Creatinine (mmol/l)	112	58	54 - 95
Potassium (mmol/l)	6.1	4.4	3.5 - 5.3
Sodium (mmol/l)	144	140	133-146
Bicarbonates (mmol/l)	7.6	23.1	22-29
Lactate (mmol/l)	13.10	0.60	0.5-2.2
Calcium (mmol/l)	1.93	2.23	2.20-2.70
NT pro-BNP (pg/mL)	51.33	917.10	125-300
Alanine transaminase (u/L)	8	6	0-55
Aspartate transaminase (u/L)	19	21	5-34
Interleukin-6 (pg/ml).	19	15	<7
Ferritin (mcg/L)	449.2	256	11-304
C-reactive protein (mg/L)	1	164	0-5

**Figure 1:**
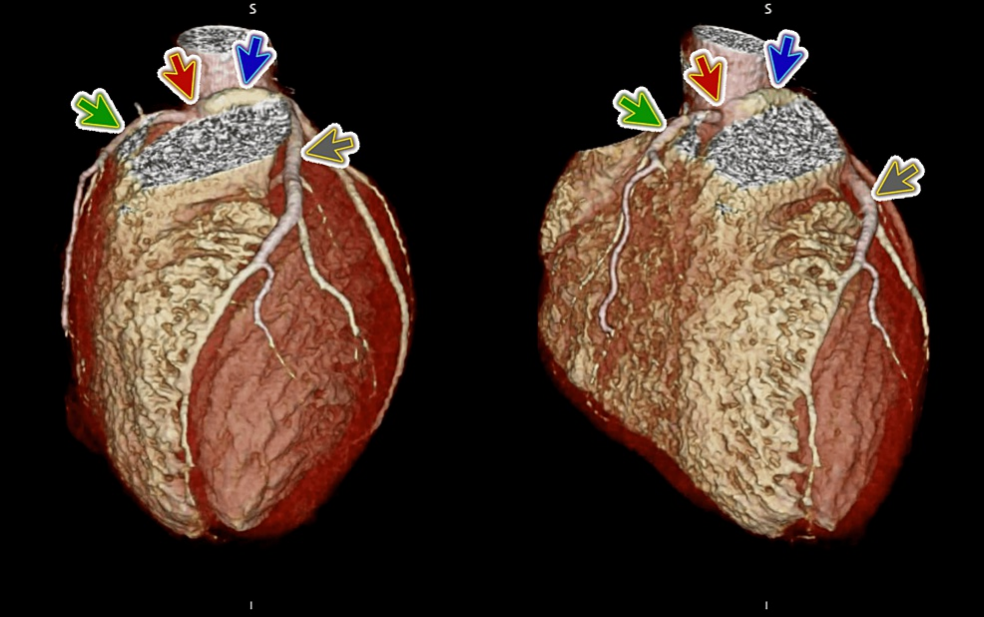
Computed tomography coronary angiography Computed tomography coronary angiography shows an anomalous origin of the left main coronary artery (blue arrow) arising from the right coronary sinus (red arrow); the grey arrow represents the left anterior descending coronary artery, and the green arrow represents the right coronary artery.

Post-cardiac arrest X-ray of the chest posterior-anterior (PA) view showed pulmonary parenchymal opacification with Kerley B lines suggestive of pulmonary edema. During the stay in MICU, diagnostic coronary angiography showed an anomalous origin of the left main coronary artery arising from the right coronary sinus (Figure 1). It also showed a malignant intraarterial course between the aorta and main pulmonary artery with minimal compression (Video 1).

**Video 1:** Computed tomography coronary angiography Computed tomography coronary angiography shows an anomalous origin of the left main coronary artery arising from the right coronary sinus and also shows a malignant intraarterial course between the aorta and main pulmonary artery with minimal compression.

Morphological cardiac MRI​​​​​​​ showed normal biventricular volumes and function. No evidence of overt myocardial edema, fibrosis, infarction, any evidence of gross cardiomyopathy, or recent myocarditis.

Repeat echocardiography after three days showed markedly improved LV systolic function compared to previous studies, ejection fraction (EF) around 45%, and normal LV diastolic function.

The patient was then referred to a higher center for surgical intervention and underwent unroofing of the left coronary artery and left translocation of the main pulmonary artery. The patient was discharged after successful surgery.

## Discussion

Cardiac arrest in young children is always a challenging scenario for the emergency department physician. Identifying a reversible cause is very important for better resuscitation outcomes. Coronary anomalies in young children represent a life-threatening form of congenital cardiac pathology. Many theories have stated that these anomalies can cause sudden cardiac death because of contortion of the vessel's slit-like tangential origin during exercise or sports activities, leading to ischemia and resultant arrhythmia. Some theories suggest that compression of an anomalous coronary artery occurs with dilatation of the aorta and pulmonary artery during exercise or sports activities [4-6].

A retrospective cohort study studied the most common cause of sudden non-traumatic death related to exercise was the cardiac abnormality, especially the coronary artery abnormalities (61%) and coronary artery anomaly (33% of all cases). Most of the anomalies were arising of the left coronary artery from the right sinus of Valsalva [7].

Yamanaka et al.'s angiographic study found that the overall incidence of coronary artery anomalies in a population of more than 120,000 patients was approximately 1.3%. The left anomalous coronary artery originating from the right sinus of Valsalva was found in only 22 patients; while this is a small proportion, the condition is serious [2]. The management of coronary artery anomalies is controversial and depends on the discovered anatomy. In terms of treatment, surgery is the mainstay, although medical management has also been used to lessen ischemic symptoms. Patients with symptoms should be treated with beta-blockers and advised to avoid strenuous physical exercises or sports as per Brothers et al. [3].

Our patient had an anomalous origin of the left coronary artery (LCA) from the right sinus of Valsalva. It had a malignant intra-arterial course between the aorta and pulmonary artery, classified as the most dangerous, placing such patients at the highest risk of sudden cardiac death.

Major society guidelines have recommended surgical intervention (class 1 recommendation) for all patients presenting regardless of ischemia or symptoms because of lack of data and inability to predict the risk of sudden cardiac death [3]. This case highlights that ED physicians should maintain a high degree of suspicion for such anomalies in case of cardiac arrest in such a young population post strenuous exercise or sport. In the event of a cardiac arrest, ED staff must put maximum effort into resuscitation, as in our case, where the patient was discharged with a normal neurological condition.

The patient underwent a successful surgery of unroofing the left coronary artery and left translocation of the main pulmonary artery. Surgery is the mainstay of treatment. Brothers et al. opine that symptomatic patients should be treated with beta-blockers and should be advised to avoid strenuous physical exertion [3]. Percutaneous or surgical treatment is reserved for anomalies with preaortic trajectories of the anomalous artery course.

## Conclusions

Coronary anomalies in young children may represent a life-threatening condition or even a cardiac arrest. The anomalous origin of the left main coronary artery from the right sinus of Valsalva is rare and can cause ischemia and resultant arrhythmia during exercise or sports activities, leading to sudden cardiac death.
